# An *in vitro* model of cancer invasion with heterogeneous ECM created with droplet microfluidics

**DOI:** 10.3389/fbioe.2023.1267021

**Published:** 2023-11-23

**Authors:** Mohammad Jouybar, Jelle J. F. Sleeboom, Elnaz Vaezzadeh, Cecilia M. Sahlgren, Jaap M. J. den Toonder

**Affiliations:** ^1^ Microsystems, Eindhoven University of Technology, Eindhoven, Netherlands; ^2^ Institute for Complex Molecular Systems, Eindhoven, Netherlands; ^3^ Soft Tissue Engineering and Mechanobiology, Eindhoven University of Technology, Eindhoven, Netherlands; ^4^ Turku Centre for Biotechnology, Åbo Akademi University, Turku, Finland

**Keywords:** cancer-on-a-chip, extracellular matrix, microfluidics, tumor invasion, heterogeneous ECM, tumor micro-environment

## Abstract

Metastasis is a multi-step process that is critically affected by cues from the tumor micro-environment (TME), such as from the extracellular matrix (ECM). The role of the ECM in the onset of metastasis, invasion, is not yet fully understood. A further complicating factor is that the ECM in the TME is mostly heterogeneous, in particular presenting a basement membrane (BM) directly enveloping the tumor, which acts as a barrier to invasion into the surrounding stromal ECM. To systematically investigate the role of ECM in invasion, appropriate *in vitro* models with control over such ECM heterogeneity are essential. We present a novel high-throughput microfluidic approach to build such a model, which enables to capture the invasion of cancer cells from the tumor, through the BM and into the stromal tissue. We used a droplet-maker device to encapsulate cells in beads of a primary hydrogel mimicking BM, Matrigel, which were then embedded in a secondary hydrogel mimicking stromal ECM, collagen I. Our technology ultimately provides control over parameters such as tissue size, cell count and type, and ECM composition and stiffness. As a proof-of-principle, we carried out a comparative study with two breast cancer cell types, and we observed typical behavior consistent with previous studies. Highly invasive MDA-MB-231 cells showed single cell invasion behavior, whereas poorly invasive MCF-7 cells physically penetrated the surrounding matrix collectively. A comparative analysis conducted between our heterogeneous model and previous models employing a single type of hydrogel, either collagen I or Matrigel, has unveiled a substantial difference in terms of cancer cell invasion distance. Our *in vitro* model resembles an *in vivo* heterogeneous cancer microenvironment and can potentially be used for high throughput studies of cancer invasion.

## 1 Introduction

Metastasis is a complex multi-step process, in which cancer cells go through invasion, intravasation, survival in the circulation and extravasation before colonizing a secondary site (Lambert et al., 2017). The onset and progression of this cascade of events are not only dependent on intrinsic (epi-)genetic factors, but they are also critically affected by biochemical, mechanical, and cellular cues from the tumor micro-environment (TME) (Sleeboom et al., 2018b). Arguably, the most critical step in the metastatic cascade is its onset, characterized by local *invasion*: Cancer cells break through the basement membrane (BM) to enter the stromal extracellular matrix (ECM).

**FIGURE 1:**
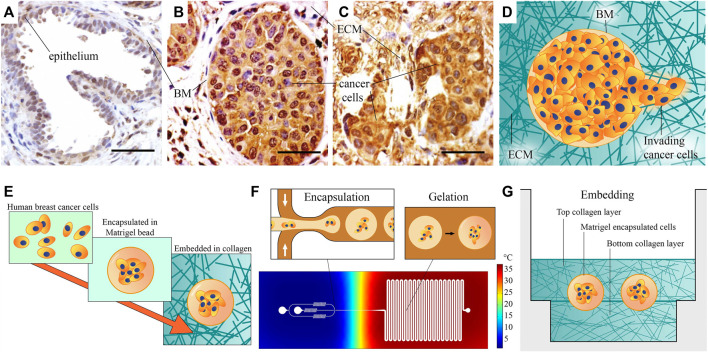
Microscopic images of healthy and cancerous breast tissue, and schematic of our method for the fabrication of the heterogeneous ECM model: **(A)** A healthy mammary gland, with intact epithelium and basement membrane (BM). **(B)** Ductal carcinoma *in situ*, or pre-invasive breast cancer. Cancer cells have filled the lumen, but the BM is still intact. **(C)** An invasive tumor, with no recognizable organization. **(A–C)** Adapted from Xu et al. (2015). All scale bars are 50 µm. **(D)** A schematic representation of invasion, showing invading cancer cells that have penetrated the BM. **(E)** Schematic overview of our fabrication approach: Human breast cancer cells are first encapsulated in Matrigel beads representing BM, then embedded in collagen I hydrogel. **(F)** Schematic overview of the encapsulation process. Cells are encapsulated in liquid Matrigel using the cold flow-focusing section in a microfluidic droplet-maker device, after which the droplets are transported to a meandering channel at elevated temperature to gelate the Matrigel. The temperature distribution in the chip is estimated based on a COMSOL Multiphysics simulation. **(G)** The model is finalized by sandwiching the Matrigel encapsulated breast cancer cells between two layers of collagen I, representing stromal matrix. This image provides a rough schematic and is not to scale; the approximate size of the beads is 100 μm, their distance to the bottom surface is 800 μm, and the thickness of the bottom Ibidi slide is 170 ± 5 µm.

The BM is a matrix layer that normally envelopes epithelial ducts, glands, and other interfaces (Figure 1A) and it is mainly comprised of collagen IV, laminins, fibronectin, and various linker proteins (Lu et al., 2012). In breast cancer, when epithelial cells become cancerous, they proliferate to fill the duct. This state, shown in Figure 1B, is referred to as *ductal carcinoma in situ (DCIS)*, or *pre-invasive breast cancer*. When invasion has taken place and DCIS has transitioned to *invasive ductal carcinoma (IDC)*, the organization of BM is lost, and cancer cells are dispersed throughout the stroma, shown in Figure 1C (images adapted from (Xu et al., 2015). The invasion process and morphology of heterogeneous ECM, as schematically depicted in Figure 1D, is very difficult to capture and study *in vivo*. An appropriate high throughput *in vitro* model could enable to study outstanding questions about invasion, such as what triggers single-cell *versus* collective invasion (Friedl et al., 2012; Ilina et al., 2020) and which role is played by ECM properties, such as the stiffness and morphology of the BM and the stromal matrix (Barcus et al., 2015; Chen et al., 2016; Hayn et al., 2020).

Many of the crucial cues in the TME are neglected when cells are cultured *in vitro* using two-dimensional models, whereas three-dimensional (3D) models can better represent *in vivo* conditions for studying ECM-cell interactions (Lee et al., 2007). ECM is a key regulator of normal homeostasis and tissue phenotype in healthy and malignant tissues, and integrating it in cellular models modulates the signaling pathways (Fata et al., 2003; Bissell et al., 2003; Bissell et al., 2005). Regulating the ECM properties in 3D models, can provide *in vivo* like conditions for studying the tumor progression. For example, M. Bissel’s group introduced a laminin-rich ECM to study the role of basement membrane in growth and differentiation patterns of normal and malignant human breast epithelial cells (Petersen et al., 1992; Lee et al., 2007). In order to investigate the role of the TME in invasion, several *in vitro* models have been developed over the past few years, based on the rapidly growing field of Cancer-on-a-Chip. In an early microfluidic study, micro-gaps between two chambers were filled with Matrigel, as a model for the BM layer, and cells were driven to invade through the gaps by chemotaxis (Chaw et al., 2007). In this model, Matrigel degradation by a single leader cell was observed. Several researchers have developed microfluidic chips which contain adjacent tumor and stromal regions, with compositions closer to *in vivo* ECM (Truong et al., 2016; Du et al., 2018; Nagaraju et al., 2018). Using these models, it is possible to form a boundary between cancer and stromal compartments, which facilitates visualization of single cell invasion, and quantitative analyses. Moreover, several approaches have been developed to investigate collective invasion from dense cell clusters. An early method, with relatively low level of control, relied on cancer cell proliferation in hydrogel to develop DCIS-like cell clusters (Sung et al., 2011). A more controlled method, used to study the effects of interstitial flow, was based on patterning of a large cancer cell aggregate with a distinct tip inside collagen I gel (Tien et al., 2012; Piotrowski-Daspit et al., 2016). Collective invasion could be observed, but only from one tumor per device, making this a relatively low throughput system. In addition, few of these studies took into account the BM that plays a major role in cancer invasion (Reuten et al., 2021).

The most complete *in vitro* models of DCIS are based on lumen patterned chips with a collagen I hydrogel matrix. They contain epithelial cancer cells, and sometimes also fibroblasts inside the collagen I matrix. Typically, these models have relatively large lumens with a diameter of about 400–500 µm. In this organotypic conformation, invasion has been studied from both DCIS (Bischel et al., 2015a) and pre-invasive pancreatic cancer (Bradney et al., 2020). In breast cancer, the presence of fibroblasts was shown to be essential for invasion (Bischel et al., 2015a). Recently, the metabolic phenotypes inside a DCIS model were found to correspond to *in vivo* DCIS phenotypes (Ayuso et al., 2018), highlighting the physiological relevance of these kinds of models. Based on an approach introduced by Lamichhane et al. (2016), Shoval et al. (2017), and Li et al. (2022) created multicellular spheroids containing, next to cancer cells, also mesenchymal stromal cells (MSC) and/or endothelial cells (EC) as another way to realize “synthetic tumor microenvironment mimics” (STEM). Using this model, Li et al. (2022) demonstrated the role of the interplay between cancer cells, stromal cells and edothelial cells in vasculature formation and in driving DCIS and IDC phenotype in migration modes of the cancer cells in Matrigel.

Although these models have shown great potential in mimicking breast cancer invasion, using them is still quite complex and labor intensive. This inhibits more widespread use and accelerated development of this promising technology. On the other hand, alternative methods with higher throughput have limitations in matrix materials, or the level of control they offer. The specific structure of BM during invasion and the effect of the surrounding matrix on the process is still a standing question that necessitates a high throughput precise model to be tackled.

In this work, we present a method to make and analyze a pre-invasive breast cancer model in a heterogeneous ECM, which addresses these issues. We aim to generate a model of pre-invasive breast cancer, without sacrificing throughput or abandoning representative matrix materials. Matrigel and collagen I are selected as models of BM and stromal ECM, due to their resemblance to these matrices. Our model is made at a scale comparable to *in vivo* breast glands, and it consists of a heterogeneous combination of the Matrigel and collagen I. We achieve this by using a high-throughput bottom-up fabrication approach, illustrated in Figure 1E. The heterogeneous TME of our pre-invasive breast cancer model is fabricated in two steps: 1) We encapsulate human breast cancer cells in Matrigel beads (Figure 1F), after which 2) we embed these in a collagen I sandwich in several micro-wells (Figure 1G). The fabrication approach is based on droplet microfluidics, which is relatively straightforward to upscale once optimized, to facilitate parallel experimentation and more widespread use. As shown in Figure 1F, we use a microfluidic chip that contains a cold flow-focusing section in which liquid Matrigel droplets encapsulating cancer cells are formed, and a warm section where the Matrigel is gelated in a meandering incubation channel to obtain Matrigel beads encapsulating cancer cells.

Two distinct types of breast cancer cells, namely MCF-7 [characterized as poorly aggressive and low-invasive, Comşa et al. (2015)] and MDA-MB-231 (recognized as highly metastatic), were employed to investigate and compare the invasion dynamics within the heterogeneous model. This selection allowed for a comprehensive examination of invasion patterns. Furthermore, a comparative study was conducted between our model, incorporating both Matrigel and collagen I, and conventional single-ECM culture models, utilizing either Matrigel or collagen I for embedding tumoroids. The behavior of cancer cells was quantified by analysis of the number of single-cell invasions and the distance of invasion across all culture conditions.

**FIGURE 2:**
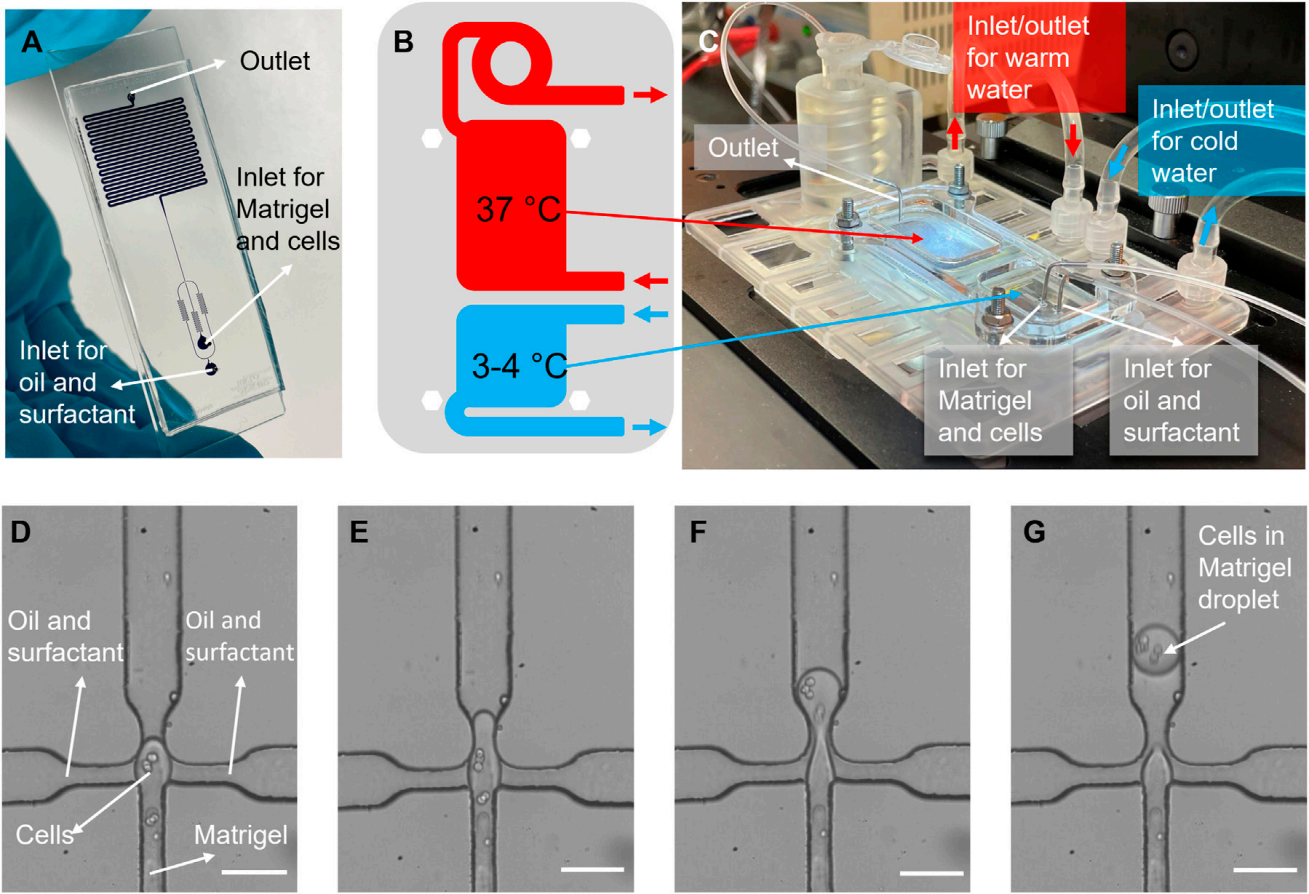
Cell encapsulation in Matrigel process. **(A)** A picture of the microfluidic encapsulation chip, filled with blue dye. The flow-focusing section is visible at the bottom, and the meandering channel section for bead gelation is visible on the top. **(B)** A schematic view of two different temperature regions, and **(C)** photo of the temperature control platform, showing how the chip is mounted onto the well-plate sized platform. Warm and iced-cold connector lines are indicated with red, and blue arrows, respectively. **(D–G)** Images of cell encapsulation in the flow-focusing junction, where Matrigel droplets encapsulating cancer cells are formed in oil. Scale bars: **(D–G)** 50 µm.

**FIGURE 3:**
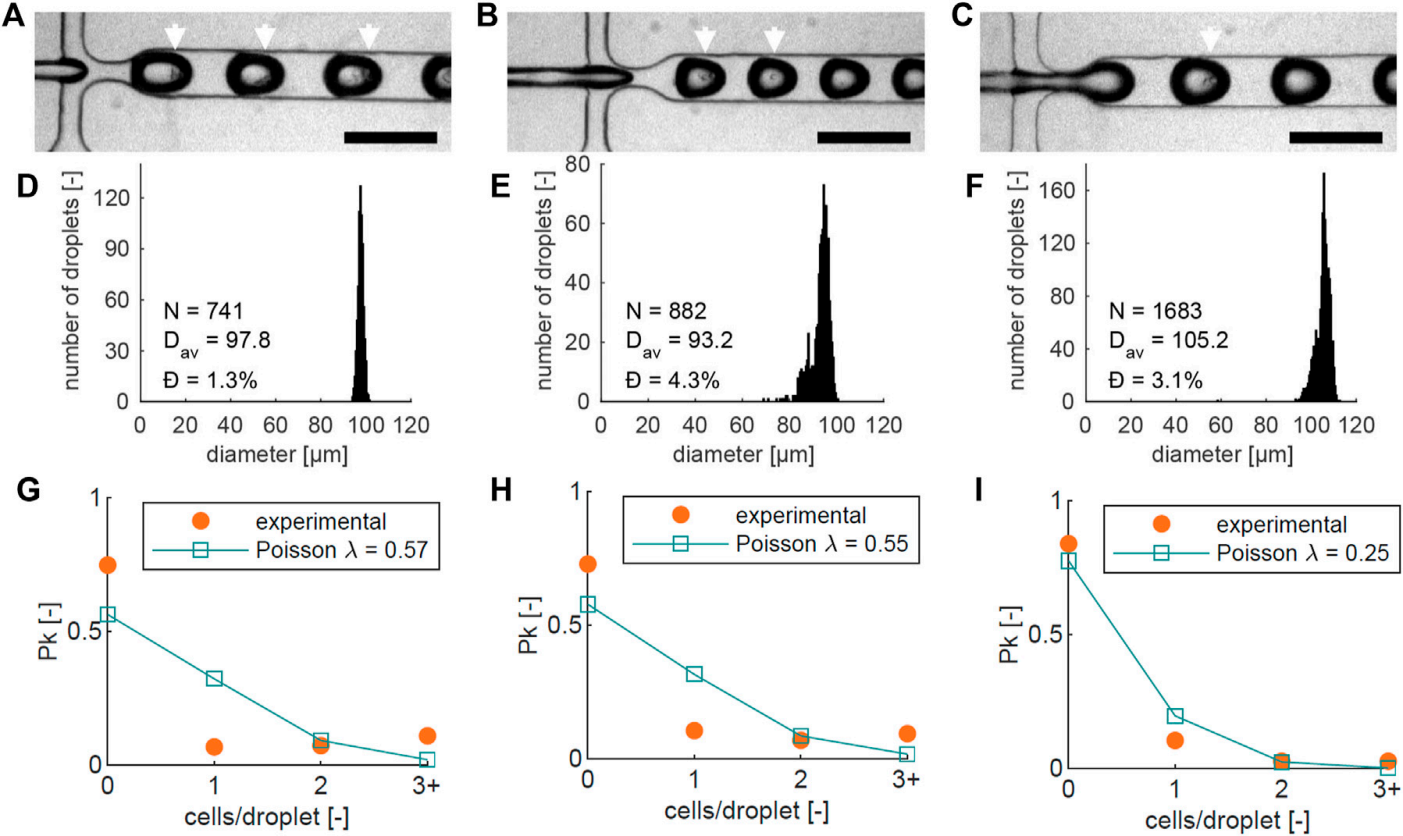
For three different chips (left, center, right): **(A–C)** Images of cell encapsulation in the flow-focusing junction, with a 200 µm scale bar, showing single cells and clusters inside the droplets. Droplets with visible cells are highlighted with white arrows; **(D–F)** Droplet diameter histograms of the three different runs in **(A–C)**. The number of droplets (*N*), average diameter (*D*_*av*_), and dispersity *Ð* are noted on each graph; **(G–I)** Graphs showing the measured proportion of beads (*P*_*k*_) containing 0, 1, 2, or more (3+) cells (orange), together with the theoretical Poisson distribution based on the cell numbers counted at the encapsulation junction in each experiment (blue). The average number of cells per bead *λ* that corresponds to the counted cell numbers is indicated in the graph.

**FIGURE 4:**
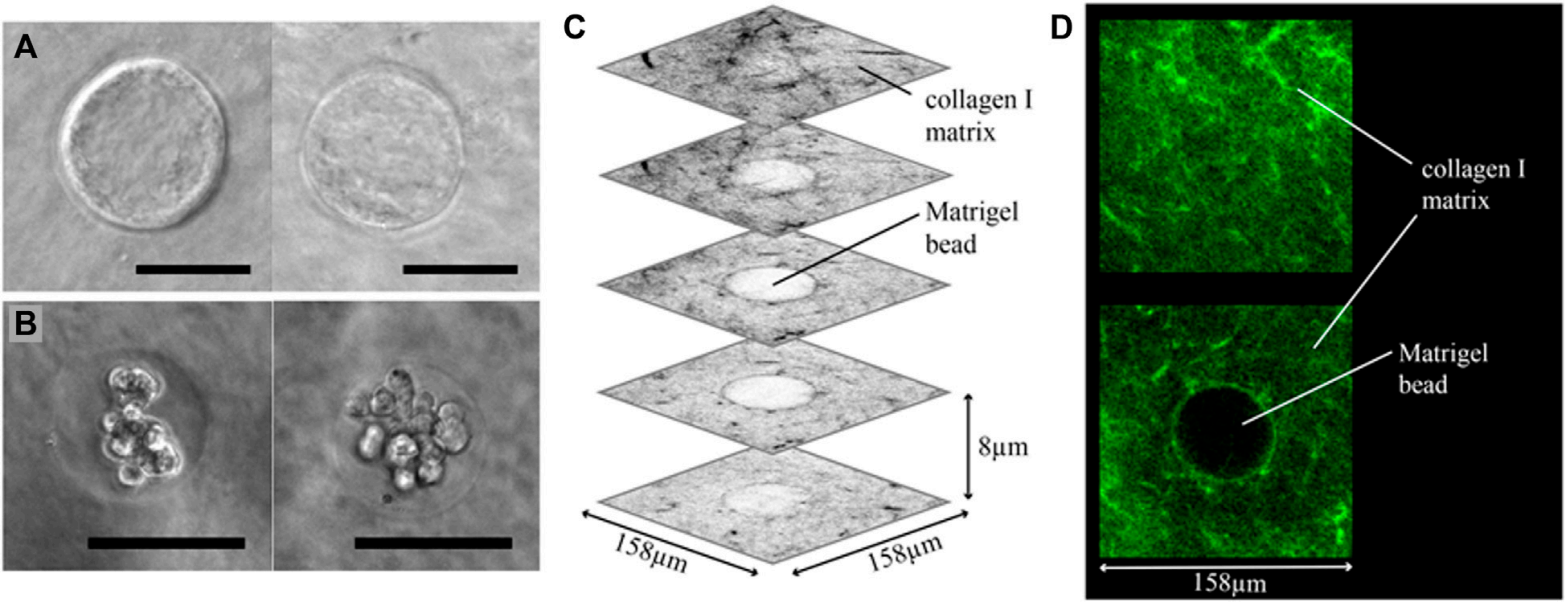
Cancer beads embedding results. **(A)** Phase contrast images of Matrigel beads embedded in a collagen I matrix. The Matrigel-collagen I boundary is clearly visible. **(B)** Matrigel encapsulated and embedded MCF-7 breast cancer cell clusters. **(C)** Several grey-scale images from a 3D confocal scan of a collagen I embedded Matrigel bead, in which collagen I is labelled with a fluorescent probe. Collagen I fibers are visible around the entire Matrigel bead, showing that the bead is fully embedded inside the collagen matrix. **(D)** Two images from the same confocal stack, clearly showing the fibrous nature of the collagen I matrix. Scale bars: **(A,B)** 50 µm.

**FIGURE 5:**
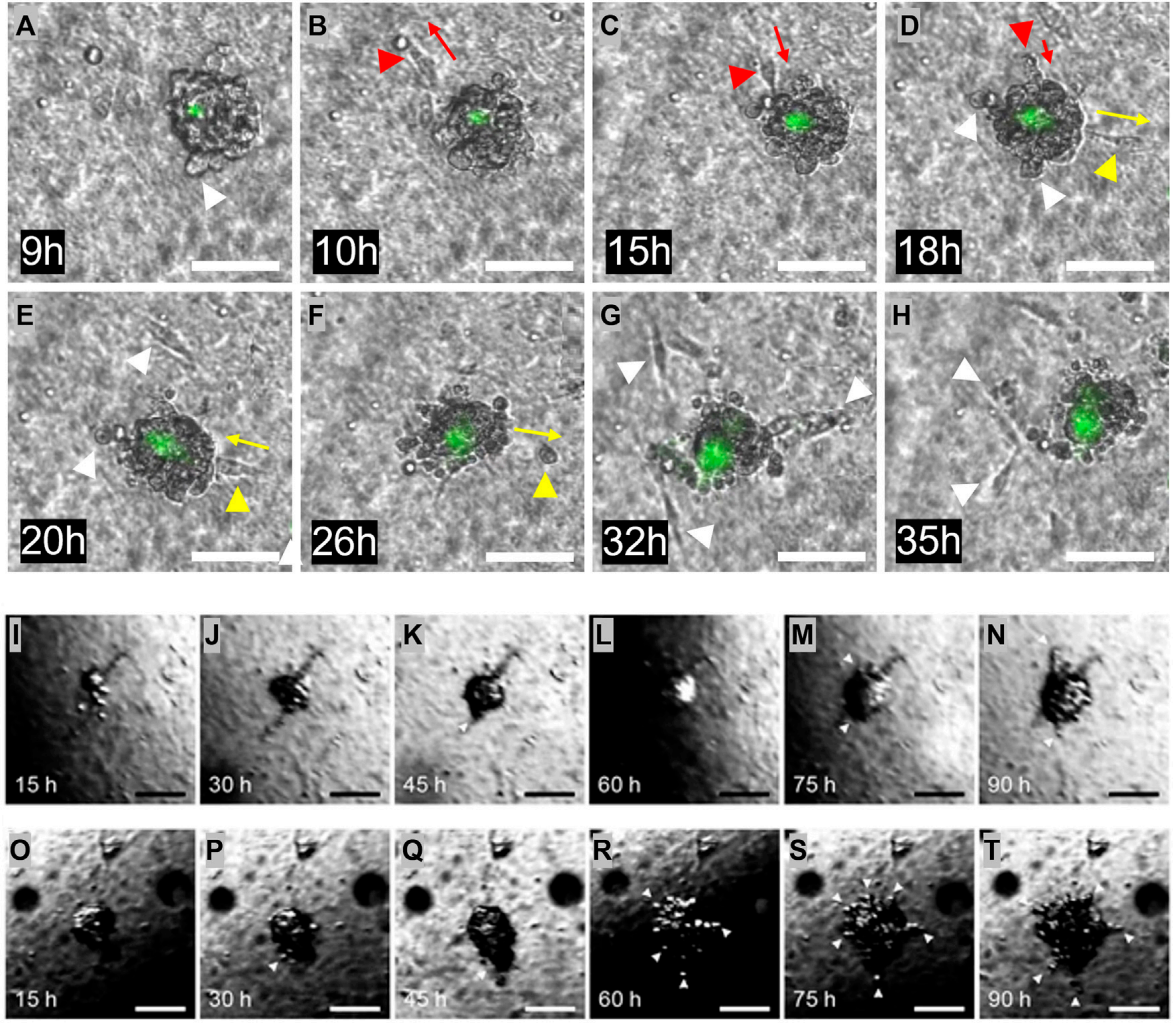
Invasion from the heterogeneous ECM. **(A–H)** One Matrigel encapsulated MDA-MB-231 cell cluster, followed during 2 days. The initial cell invasion into the surrounding collagen I was seen after 9 h. Invading cells are highlighted with white arrows. Multiple forth and back single cell movements were seen, indicated with red and yellow arrows. Dead cells are stained in green. **(I–N)** One Matrigel encapsulated MCF-7 cell cluster, followed during 4 days. During the first 2 days, the cells proliferate and fill the Matrigel capsule. After this time, cells start to penetrate the Matrigel and move into the surrounding collagen. Invading cells are highlighted with white arrows. **(O–T)** Another Matrigel encapsulated MCF-7 cell cluster followed during 4 days, with more erratic behavior. The Matrigel bead is already full of cells after 15 h, and some penetration is seen already after 30 h. Penetrating cells are highlighted with white arrows. The dark region seen in some of the images is caused by refraction of light at the medium surface. Scale bars: **(A–T)** 100 µm.

**FIGURE 6:**
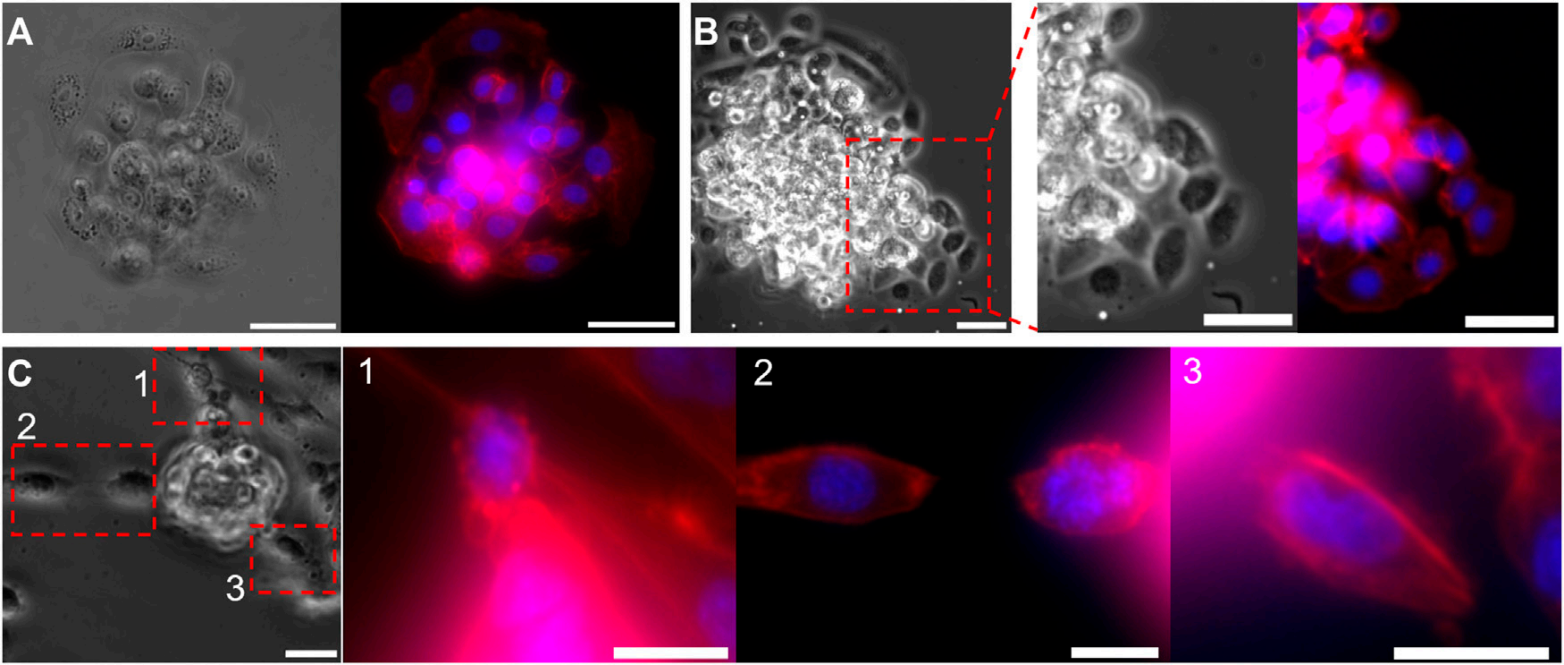
Invasive morphology of two types of cancer cells, from a micro-tumor generated with droplet microfluidics. **(A,B)** Bright-field and fluorescent images of the MCF-7 cell cluster bead, **(A)** before and **(B)** after breaking through the Matrigel. Focused images in **(B)**, show the collective invasion behavior of MCF7 cells. **(C)** Bright-field and fluorescent images of a MDA-MB-231 cell cluster bead during invasion. Focused fluoresent images of the regions 1,2, and 3, shows single cell invasion. Cell nuclei are stained in Blue and F-actin in red. Scale bars: **(A,B)** 50 μm, **(C)** 25 µm.

**FIGURE 7:**
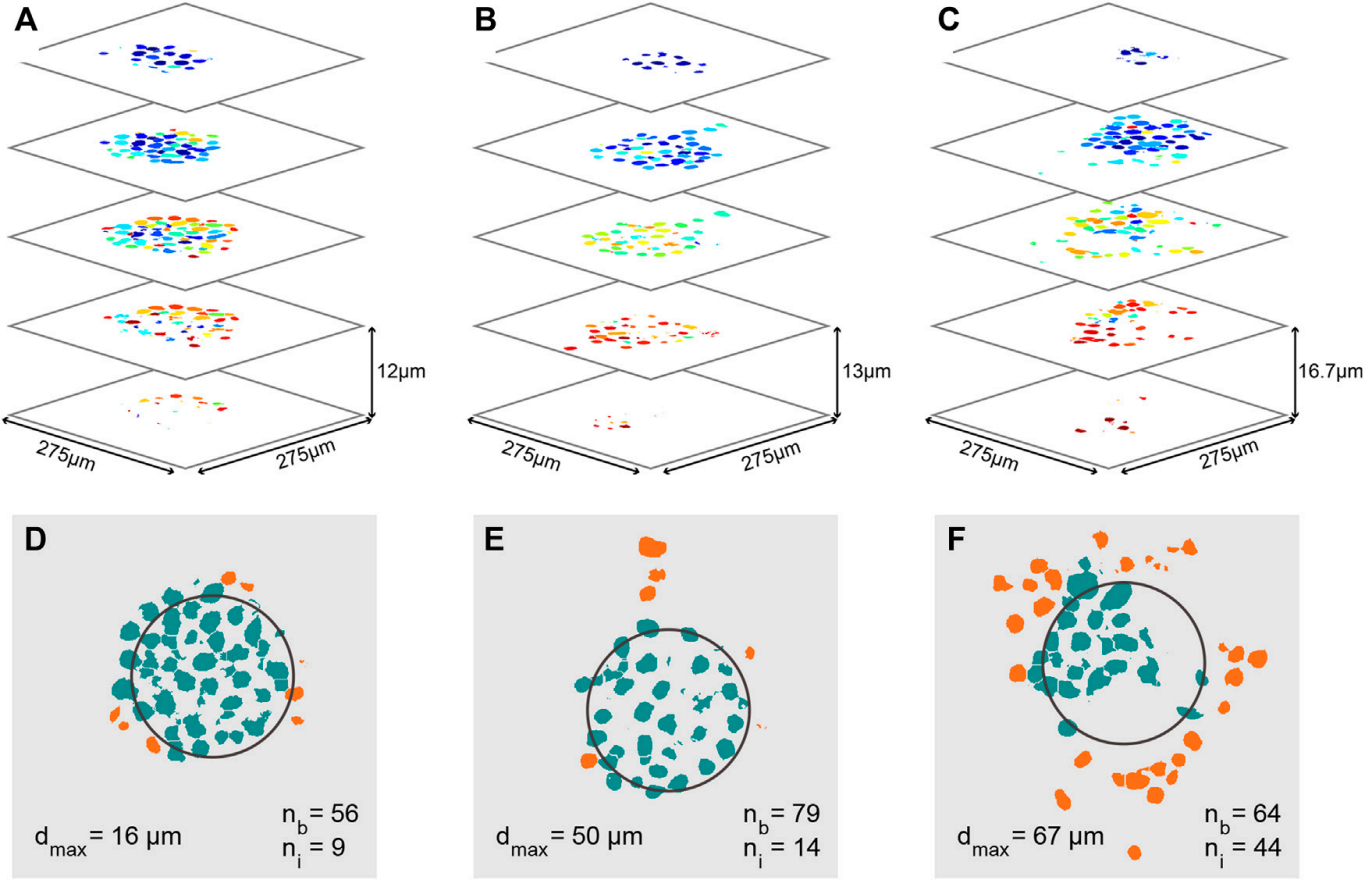
Quantitative analysis of cancer invasion in the heterogeneous ECM model. **(A–C)** Analyzed confocal images of stained nuclei from MCF-7 microtumors after automatic post processing for cell counting. Images of several slices of three microtumors are shown; the cells in **(A)** did not invade as much as the ones in **(B,C)**. **(B,C)** Images of several slices of the invaded microtumor from Figures 5I–T, respectively. Each individual cell nucleus is colored differently. In the three stacks, a total cell number of 65, 93, and 108 was counted respectively. **(D–F)** Slices through the center of each microtumor in **(A–C)**, in which the approximate original position of the Matrigel bead is outlined in black. The number of cells still inside the bead (*n*_*b*_), the number of invaded cells (*n*_*i*_), and the maximum invasion distance (*d*_max_) are indicated on the images. Cells in the bead are colored green and cells that have invaded are orange.

**FIGURE 8:**
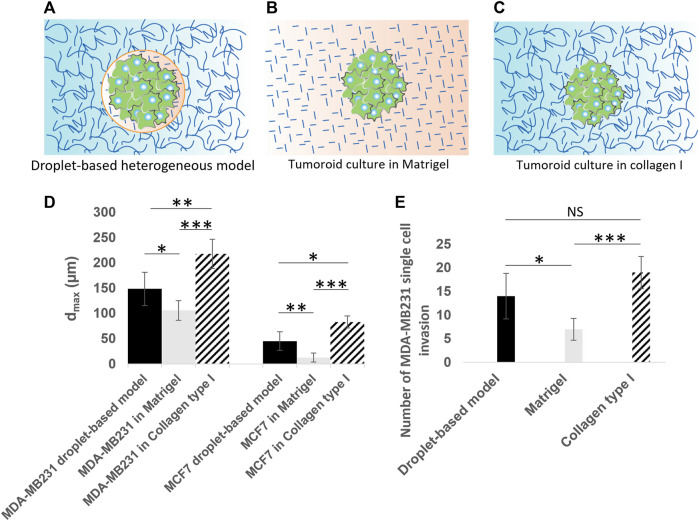
Quantitative analysis of cancer invasion in the heterogeneous ECM model compared to single-ECM models. **(A–C)** Schematic of the three culture conditions being **(A)** a tumoroid in our droplet-based heterogeneous ECM, **(B)** a tumoroid in Matrigel, and **(C)** a tumoroid in collagen I. Cancer cells are colored in green. Collagen I is shown with long curved lines, and Matrigel with short straight lines. **(D)** The maximum invasion distance measured for MCF-7 and MDA-MB-231 cells in three culture conditions, including the heterogeneous model, Matrigel only and collagen I only. **(E)** Number of single MDA-MB-231 cell invasions from tumoroids cultured in the heterogeneous droplet-based model, Matrigel only, and collagen I only. *p < 0.05, **p < 0.01, and ***p < 0.001 indicate statistical significance. NS, not significant. Two-tailed Student’s *t*-test was done to determine statistical significance.

In summary, we present a novel engineering technique for creating a heterogeneous ECM (Figure 1). Our approach involves the utilization of a droplet-maker system integrated within a microfluidic chip, allowing for the maintenance of two distinct temperatures (Figure 2). We can control the diameter and number of the generated droplets to ensure optimal conditions for subsequent experiments (Figure 3). We closely monitor and analyze the invasion behavior of two different types of cancer cells within our developed model (Figures 4–6). Finally, we perform a comparative analysis on the invasion behavior of cancer cells in our heterogeneous ECM model with conventional single-ECM models commonly used in the field (Figures 7, 8).

## 2 Materials and methods

### 2.1 Encapsulation device fabrication

The microfluidic chip was fabricated using standard soft lithography methods. A mold was produced by first spin-coating silicon wafer (Si-Mat) with a photoresist (Microchem, SU-8 3050) according to manufacturer’s protocol, to obtain a final layer thickness of 100 µm corresponding to the channel height. After a soft-bake at 95°C for 50 min, the wafer was covered with a photomask (CAD/Art Services, Inc.) containing the channel design and exposed to UV-light for 18 s at 14 mW/cm^2^. After post exposure bake (first, 65°C for 1 min and then at 95°C for 5 min), the wafer was placed in developer (micro resist technology GmbH, mr-Dev 600) on an orbital shaker for 15 min, then rinsed with isopropanol and ethanol, and dried with a Nitrogen jet. For the purpose of mass production, the replication of the wafer was achieved through the utilization of epoxy molding, as described previously (Ballerini et al., 2022).

PDMS devices were fabricated by mixing Sylgard^®^ 184 PDMS base with Sylgard^®^ 184 curing agent (both from Merck) at a 10:1 w/w ratio, cast onto the molds, and degassed for 1 h. After curing the PDMS at 65°C for at least 3 h, the PDMS slabs were peeled off from the molds, and in- and outlet holes were made using a 1.2 mm biopsy puncher (VWR, WHATWB100074). The channels were sealed by bonding the PDMS slab to previously prepared PDMS-coated microscope slides. These slides were made by spin-coating PDMS on a microscope slide (VWR, 631-1552) at 4,000 rpm for 40 s, and curing them at 65°C for over 3 h. The PDMS slabs and glass slides were both exposed to 50 W air plasma for 45 s using a plasma asher (Emitech, K1050X), and brought into contact. After bonding, the channels were treated with 5% perfluorooctyltriethoxysilane in HFE-7500 (both from Fluorochem), incubated for 1 h at 65°C, flushed with HFE-7500, and incubated overnight at 65°C for thermal bonding.

### 2.2 Fabrication of a temperature controller system

The temperature control platform design was made in Siemens NX (Siemens AG) and then transferred to PreForm software (Formlabs). A durable resin cartridge was inserted into a Low Force Stereolithography 3D printer (both from Formlabs), and the printing was run. When the print was complete, the platform was placed in Form-Wash (Formlabs) for 30 min, in order to wash the uncured resin in isopropyl alcohol, and the resin then was cured in Form-Cure (Formlabs) for 1 h (the design is provided in Supplementary Figure S1). Glass microscopy slides were attached to the 3D printed platform using Dowsil™732 silicon glue (Dow Corning) to seal off the water chamber. The cooling chamber was connected via tubing (Tygon, E3603) to a positive displacement water pump (7026898, RS PRO), pumping ice water. The warm chamber was connected via tubing to the same type of pump, for which the water source was placed on a heater. The heater temperature was set to 41°C which resulted in a temperature of 37°C in the chamber.

### 2.3 Temperature control simulation

A COMSOL finite element simulation was performed to estimate the temperature distribution in the chip. The model included the PDMS layer of the chip, its glass bottom, the water chambers, and the cooling and heating water flow. Inlet temperatures in the cold (T_c_) and warm (T_h_) chamber were estimated to be 1°C and 37°C for the cold and hot water. The environmental temperature (T_∞_) was set to 20°C. Natural convection was modelled at the top and sides of the PDMS, and at the bottom of the device holder. The steady state solution was computed, and the surface temperature of the glass chip bottom was taken as the temperature of the microchannels. For PDMS, the density, heat capacity, and thermal conductivity values were obtained from literature Yi et al. (2014). All other material properties were obtained from the COMSOL material library. The impact of the microfluidic channel flow was neglected, as the amount of heat transported via this flow is 5 orders of magnitude lower than in the cooling and heating water flow. Additionally, the temperature in the microfluidic channels was assumed to be equal to that of the PDMS.

### 2.4 Cell culture

MCF-7 and MDA-MB-231 (Sigma-Aldrich) breast cancer cells were used between passage 22 and 40. The culture medium contained RPMI-1640 (Gibco, 61870010), supplemented with 10 vol% Fetal Bovine Serum (FBS) (Bovogen, SFBS) and 1 vol% Penicillin/Streptomycin Solution (P/S) (Sanbio, SCC0503). MCF-10A cells were used between passage 5–15. The culture medium contained DMEM-F12 (Merck, D0697) supplemented with 20 ng/mL human epidermal growth factor (hEGF) (Merck, 6225363-8), 0.5 μL/mL Hydrocortisone (Merck, 50-23-7), 100 ng/mL cholera toxin (Merck, 9012639), 10 μg/mL insulin (Merck, 11061680), 5% horse serum (Merck, H1270) and 1 vol% Penicillin/Streptomycin Solution (P/S) (Sanbio, SCC0503).The cells were kept in T75 cell culture flasks at 37°C and 5% CO_2_, and passed in a 1:10 ratio when they reached 70%–80% confluence. Medium was refreshed every 2–3 days.

### 2.5 Production of hydrogel droplets

Cells were obtained during passaging, their density was determined using a cell counter (Chemometec, Nucleo-Counter NC-3000), and adjusted to 2.5 ⋅ 10^7^ cells/mL. The cells were encapsulated in Corning growth factor reduced Matrigel (VWR, 734-0269). All subsequent steps were done on ice and with pre-cooled equipment to prevent early Matrigel gelation. A final cell density of 2.5 ⋅ 10^6^ cells/mL was reached by mixing 15 µL of cell suspension with 135 µL of thawed Matrigel. The hydrogel droplets were generated using the droplet maker chip with two inlets and one outlet. The chip was placed on the temperature controller system, and 100 µL of the Matrigel-cell suspension was injected in the chip inlet tube, which was continuously cooled by a surrounding water-flow ( < 4°C) provided by an ice water reservoir. The heating water was provided from a water reservoir on top of a hot-plate, set to 41°C. Next, the innermost inlet tube was connected to a pre-filled syringe with mineral oil (Sigma, M5310) on a pumping system (Cetoni, neMESYS low pressure syringe pump), and set to a flow-rate of 1 μL/min (Matrigel channel). Fluorinated oil HFE-7500 (Fluorochem BV) with 2.5% surfactant PicoSurf (SphereFluidics) was flushed into the chip via the other inlet connected to the outer channels of the droplet-making flow-focusing section (see Figure 1F) at a flowrate of 30 μL/min (oil channel). These flow rates resulted in generated droplets with a diameter of 100 µm. After creation of the droplets, they entered into the warm meandering channel to gelate. The created bead emulsion in fluorinated oil was collected from the outlet in an Eppendorf tube. The Eppendorf tube was located in the warm holder of the temperature control system; the residence time of beads in the meandering channel and Eppendorf tube was 15 min. The Eppendorf tube was then placed in an incubator for 30 min to ensure the polymerization of the hydrogel droplets before further use.

### 2.6 Bead retrieval from fluorinated oil

After collecting the emulsion of Matrigel beads in fluorinated oil, the oil remained at the bottom of the vial due to its high density (HFE-7500 has a density ≈1.6 g/mL), while the Matrigel beads floated on the top oil surface. The oil [including PicoSurf (SphereFluidics)] was aspirated from the bottom of the Eppendorf collection tube until the floating beads reach the bottom. 200 μL of HFE3500 oil (3M™ Novec™ 7500 Engineered Fluid) was added to the tube to dilute the residue of surfactant. A hydrophobic paper filter (Sigma-Aldrich, WHA2200070) was used to eliminate the oil from the emulsion. 20 μL of floating beads were aspirated from the top of the emulsion, and unloaded on the hydrophobic filter, upon which the oil was absorbed by the filter. Afterwards, 20 µL of RPMI cell media was added on top of the filter. The hydrophobic paper filtering was repeated 3–4 times by transferring the solution onto fresh papers to remove the oil completely. The bead suspension was then transferred to a fresh collection tube. Finally, beads suspension in media were centrifuged (900 rpm, 2 min) to sediment the beads at the bottom of the collection tube. 15 μL of media was removed from the top and the remaining 5 µL of bead suspension was aspirated for use in the experiments.

### 2.7 Matrigel beads embedding in collagen I

Cell containing beads were embedded in collagen I by sandwiching them between two collagen layers, in Ibidi µ-slides (Ibidi, 81506) that enabled the formation of a flat bottom hydrogel layer, shown schematically in Figure 1G. A 1.5 mg/mL collagen I hydrogel (Gibco, A10483-01) was prepared on ice per the manufacturer’s instructions. A 10 µL bottom gel layer was pipetted into each µ-slide well, and incubated at 37°C for 30 min for gelation. 5 μL of cell encapsulated bead was aspirated and deposited on collagen I layers in each well. Next, a second layer of collagen I (20 µL) was deposited in each well. After another 30 min incubation step, medium was added to the wells, and the slide was placed in an incubator at 37°C and 5% CO_2_. Cell medium was refreshed every day. Samples were fixed after experimental time, which was 2–4 days depending on cell type.

### 2.8 Production of hydrogel beads using mineral oil and their retrieval from oil

In initial phases of experiments, we used mineral oil (Sigma, M5310) instead of fluorinated oil. Both conditions were suitable for droplet making, however we chose fluorinated oil at the end because of its biocompatibility and easier bead recovery procedure. For mineral oil (with 4 wt% ABIL EM 90 surfactant (Evonik)), the outer phase flow-rate was lower, at 2 uL/min. For recovery of beads, 30 µL of medium was added to each well after generation of the bottom collagen I layer. Next, 10 µL of encapsulated cell suspension was added, and as well as 20 µL of mineral oil to dilute the surfactant. Next, hydrophobic filter paper (Sigma-Aldrich, WHA2200070) was placed directly on top of the wells, to absorb the oil and force the beads into the medium to sediment on the gel bottom. The paper was refreshed until all oil was removed.

### 2.9 Spheroid making for non-heterogeneous models

MCF-7, MCF-10A, MDA-MB-231 cells were diluted to 10e4 cells/mL in the cell media [RPMI-1640 (Gibco, 61870010), supplemented with 10 vol% Fetal Bovine Serum (FBS) (Bovogen, SFBS) and 1 vol% Penicillin/Streptomycin Solution (P/S) (Sanbio, SCC0503)] supplemented with MethoCel (Merck, 94378). Concentration of 2% stock MethoCel (1.2% w/v) was used for MCF-7 and MCF-10A, and of 20% stock MethoCel (1.2% w/v) for the MDA-MB-231 cells. 200 μL of this solution was deposited in each well of a round-bottom 96 well-plate (Merck, M3562), and incubated for 24 h to generate tumoroids. 10e4 cells/mL concentration led to the tumoroids with the dimension of 100 µm. The tumoroids were all transferred to a 2 mL eppendorf tube by aspirating 20 µL from the bottom of each well. The tube was centrifuged (900 rpm, 2 min) to sediment tumoroids at the bottom of tube. 10 μL of either Matrigel or collagen I was deposited in Ibidi µ-slides (Ibidi, 81506) to form a first layer of gel. After leaving the samples in the incubator for 30 min, 5 µL (1,000 tumoroids/mL) of tumoroids was dispensed on top of the first layer. Afterwards, a second 20 µL layer of either Matrigel or collagen I was deposited in each well, to complete a sandwich of tumoroids in between two layers. After incubation for 30 min, the well was supplemented with cell medium; cell medium was refreshed every day. Samples were incubated inside the incubator for 2 days, and were then fixed for further analysis. Some samples were fixed after 3 days for comparative analysis.

### 2.10 Fixation, staining, and live imaging

The cell samples were fixed using a 1.85 vol% formaldehyde (Merck, 1040031000) and 1 vol% glutaraldehyde (GA) (Sigma-Aldrich, G5882) solution in PBS (Westburg, LO BE02-017F) for 15 min. GA was added to prevent Matrigel depolymerization, as advised by the manufacturer. The samples were permeabilized by exposing them to 0.5 vol% Triton X-100 (Merck, 108603) solution in PBS for 15 min. Samples were blocked via exposure to blocking solution (1% Bovine Serum Albumin) (BSA) (Sigma-Aldrich, 9048-468), and incubated at room temperature for 1 h. The cell nuclei were stained using NucBlue Fixed Cell ReadyProbe Reagent (Thermo Fisher, R37606) and the cytoskeleton was stained for F-actin using ActinGreen 488 ReadyProbes Reagent (Thermo Fisher, R37110). Staining was performed by exposing the samples to PBS containing 2 drops/mL of each reagent for 60 min. In live imaging assays, one drop/mL of ReadyProbe NucGreen^®^ Dead reagent (Thermo Fisher, R37609) was added to the cell media to stain the dead cells. Before and after each of the previous steps, the sample was washed three times in PBS. Pure collagen I samples were not fixed, but labelled directly using a CNA35-OG probe, as previously described (Krahn et al., 2006).

### 2.11 Imaging and analysis

Several cell encapsulation videos of 10 s were captured at 30 fps using an inverted microscope (WPI, INV-101), fitted with a USB camera (The Imaging Source, 31AU03). Bead diameters were measured in these videos, using the imfindcircles function in Matlab, which is based on the Circular Hough Transform. In order to prevent double counting, droplets were tracked and counted only when they crossed the center of the frame. For more detailed analysis, a high speed camera (Fastec IL5) was used to record the droplet making process. Live-cell images were obtained using an incubator-proof camera that took pictures every 15 min (Cytosmart, Lux2 or Lux3 10x). Phase contrast, bright-field, and fluorescent images were obtained using either a Thunder imaging system (Leica Microsystems, DMi8), equipped with the LAS software or a confocal microscope (Zeiss, LSM510 META NLO).

Cell positions were automatically measured in the 3D confocal images, based on the position of cell nuclei. To locate the nuclei of individual cells, the images were segmented using Matlab code based on the 3D watershed algorithm. Linear interpolation was used to make this method suitable for the 3D confocal image stacks, which have low resolution in z-direction relative to the x-y plane: In order to avoid a bias towards x-y segmentation, the resolution in z-direction was artificially inflated by adding linearly interpolated images in the stack. The stack was then filtered, and converted to black and white by thresholding based on Otsu’s method. The image was then converted to an Euclidean distance map, on which the watershed algorithm was performed. The size of identified regions was checked, and split if it was over 2 times the average size. The bead position was estimated based on the average nucleus position, based on the centroid of each region. Individual nuclei positions were offset to the center of the estimated bead position, and their radius was estimated based on the volume of a sphere (*V* = 4/3*πr* ^3^). Cells were labelled “invaded” if their distance from the bead was more than their estimated radius.

To perform the comparative analysis on the maximum invasion distance between our heterogeneous and conventional single-ECM models, we fixed the cells after 3 days in heterogeneous model, and after 2 days in single-ECM models. The extra 1 day in the heterogeneous model was enough for cells to fill in the 100 µm bead. For the collective invasion measurements, we considered the protrusions distance from the boundary of the beads or tumoroids. MCF-7 tumoroids in Matrigel grew in time with no invasive behavior (Supplementary Figures S4E–H); this is consistent with other invasion assays using MCF-7 spheroids in Matrigel, such as reported in Li et al. (2022), and we did not consider this as collective invasion.

## 3 Results

### 3.1 A microfluidic technique for the encapsulation of cancer cells in Matrigel beads

Cell encapsulation in Matrigel beads is achieved in a system comprised of two parts: A microfluidic chip, shown in Figure 2A, and a chip-holder with integrated heat exchange chambers for temperature regulation, shown in Figures 2B, C. The microfluidic chip contains two inlets, one for the continuous phase (oil and surfactant) and one for a liquid Matrigel-cell mixture, which is emulsified in the continuous phase in a 40 × 100 µm flow-focusing junction (Figures 2D–G). Flow-rates can be tuned to produce droplets with a diameter between 40 and 100 μm, within the size range of mammary alveoli [between 25 and 200 µm (Negin Mortazavi et al., 2015)].

The chip-holder has two temperature regions that are generated by continuously pumping cool and warm water through the chambers below the chip, shown in Figures 2B, C. In order to prevent premature Matrigel gelation, which occurs at temperatures above 4°C, the inlets and junction are locally cooled since they are aligned with the cold region on the chip. Directly after droplet generation, they are transported to a 500 µm wide meandering channel, which is kept at a temperature of approximately 37°C. During the residence time (15 min) in the warm meandering channel section and collection tube, the Matrigel gelates, transforming the droplets into beads. Using the encapsulation platform, cells were successfully encapsulated in Matrigel beads. Cells in Matrigel droplets can clearly be identified in stills from videos of the encapsulation process, shown in Figures 2D–G.

### 3.2 Matrigel bead dimensions and cell encapsulation probability

Three separate cell encapsulation runs using different chips were recorded, shown in Figures 3A–C. As expected, not all Matrigel droplets contain cells, and some droplets contain multiple cells. Some size variation is present between the three experiments, which could be caused by slight variations in temperature and viscosity. To verify the bead dimensions, diameters were measured in the downstream meandering channel section. Bead diameter histograms from the three experiments are shown in Figures 3D–F, based on 741, 882, and 1683 measured droplets respectively. The average diameters (*D*_*av*_) were 97.8, 93.2, and 105.2 µm, and the dispersity *Ð*, defined as the standard deviation of the size distribution divided by the mean bead diameter (Christopher and Anna, 2007), was less than 5% in all cases: with values of 1.3%, 4.3%, and 3.1% respectively. This indicates that the beads have a narrow size distribution, close to the desired diameter of 100 µm.

The probability of a droplet containing *k* = 0, 1, 2, … cells is described by the Poisson distribution in Eq. 1 (Velasco et al., 2012), where *P*_*k*_ is the fraction of droplets containing *k* cells, and *λ* is the average number of cells per generated droplet. The number of cells per bead was analyzed by counting the proportion of beads containing 0, 1, 2, or more cells, shown in Figures 3G–I. In all three Figures, the theoretical Poisson distribution (Eq. (1)) based on the cell number counted at the junction is also plotted.

$$P_{k}=\frac{\lambda^{k}e^{-\lambda }}{k!}$$ (1)

There are small differences between the theoretical curve in all three experiments: The number of beads with 3 or more cells is higher than predicted, while the number of beads with single cells is lower. We attribute this to cell aggregation in the suspension before encapsulation, which increases the chances of encapsulating multiple cells in one droplet, and decreases the number of single encapsulated cells. It is most apparent in Figures 3G, H, where the number of beads with 3 or more cells is higher than, or equal to the number of beads with a single cell. Additionally, almost all beads with multiple cells contained one or two clusters (Supplementary Figure S2).

Aggregation reduces the number of beads containing cells, which reduces the number of beads that are available for developing potential invasion. However, it also increases the chances of microtumor formation from the beads that do contain cells, as this process is more likely to succeed with multiple initial cells [only around 20% of the MCF-7 cell population is able to form a tumor *in vitro* starting from a single cell (Calvet et al., 2014)]. Between 16% and 27% of the beads contain one or more cells, which can be the basis of an invasive cluster. Since the bead formation is a high-throughput process (Approximately 1800 beads per minute), the absolute number of beads containing cells is still high (288–486 beads containing cells per minute).

### 3.3 Reconstruction of a heterogeneous ECM

After fabrication of the Matrigel beads encapsulating cells, they are retrieved from the oil phase, transferred to cell culture medium, and embedded in collagen I. After depositing the Matrigel beads on the bottom layer, a top layer of collagen I is cast over the beads, as shown schematically in Figure 1G. Bead embedding was first tested using beads without any encapsulated cells. In Figure 4A, two phase contrast images of embedded Matrigel beads are shown, and in both images the Matrigel-collagen I boundary is clearly visible. Much like the empty Matrigel bead, beads containing cells can be embedded, as shown in Figure 4B; the state shown here is the starting point of our invasion assay, the results of which are presented in the next section.

In order to confirm that the beads were fully enveloped in collagen I, the collagen I was fluorescently labelled with a CNA35-OG probe and imaged using a confocal microscope. Several slices of a 3D image stack are shown in Figure 4C; and two slices, one with only collagen I, and a cross-section at the middle height of the bead, are shown in Figure 4D. Double encapsulation of cancer cells first in Matrigel and then in collagen I was successfully achieved, enabling the cancer invasion assay in a heterogeneous ECM.

### 3.4 Invasive behaviour of cancer cells in heterogeneous ECM

The samples containing the embedded and encapsulated MDA-MB-231 or MCF-7 cells were cultured for several days, during which microscopic images were taken every 15 min, as shown in Figure 5. Figures 5A–H are images of a developing MDA-MB-231 bead. During the first 6 h of culture, the MDA-MB-231 cells proliferated to arrange themselves into a spherical shape within the Matrigel, filling the entire bead. Although the Matrigel bead can not easily be identified in these images, the cells arrange themselves into a spherical shape with a diameter of around 100 μm, comparable to the size of the enveloping Matrigel bead. For about 3 h, the cells remained in this conformation, seemingly having arrested their growth into a microtumor. This is the DCIS state that is visible in Figure 5A. However, MDA-MB-231 cells started to gradually invade into the surrounding collagen I matrix after 9 h turning to an IDC state, exhibiting single cell invasion, as indicated with white arrows in Figures 5A–H. During this process, some cells moved away from, and then back toward the microtumor, as indicated for one cell by the red arrows in Figures 5B–D, and for another cell by the yellow arrows in Figures 5D–F. This suggests that the motion is not fully directional. This may be due to the absence of imposed gradients (such as of oxygen) or alignment of ECM collagen I fibers; such effects can be included in our model in the future. Also visible in Figures 5A–H are dead MDA-MB-231 cells stained in green. The region of dead cells is mostly limited to the core of tumor bead, and it slightly grows over time along with continuous microtumor growth and invasion development. The appearance of a dead core within spheroids/tumoroids is commonly seen in 3D culture in hydrogels, and it is attributed to a lack of oxygen and/or nutrients in the core.

Images of the development of one MCF-7 bead that contained several cells are shown in Figures 5I–N. During the first 2 days, until 35 h, the MCF-7 cells proliferated to fill the entire Matrigel bead, similar to the MDA-MB 231 cells. This can be seen in Figures 5I, J. For approximately 10 h, the cells remain in this DCIS conformation, and then cells start to break through the Matrigel-collagen I interface and slowly penetrate the surrounding collagen I matrix, indicated with arrows in the figure; this IDC state is first seen in 5K. The behavior of the MCF-7 cells is clearly different from that of the MDA-MB-231 cells: a single “leader” cell penetrates the Matrigel-collagen I interface and migrates away from the microtumor, followed by a train of other cells, similar to multicellular streaming (Friedl et al., 2012) or collective invasion (Ilina et al., 2020). This process seems to be driven by the MCF-7 cells pushing against the matrix and physically penetrating the surrounding collagen-I, since the invasive front does not show active migratory phenotype with the leader cell exposing filopodia, as in the common definition of collective invasion (Krakhmal et al., 2015). This process can be observed in the Supplementary Video S1. Another MCF-7 bead was also followed over time, shown in Figures 5O–T. This sample showed a slightly different progression, starting with an almost full bead early in the experiment. Much like the microtumor in Figures 5I–N, penetration happened after the bead was completely filled with the proliferating MCF-7 cells. For this sample, this process started around 24 h after embedding, with more cells penetrating during the remaining 3 days. Initially, this occurred in a similar collective fashion as the previous sample, but eventually cells invaded from all sides of the microtumor.

After invasion assays were completed, the samples were fixed and stained for cell nuclei, and cytoskeletal protein F-actin to visualize cell shape. Bright-field (BF) and fluorescent images of MCF-7 tumoroids before Matrigel penetration are shown in Figure 6A, and after invasion in Figure 6B. After entirely filling up the Matrigel bead, MCF-7 cells start to migrate collectively into the surrounding collagen I matrix. Magnified BF and fluorescent images of an invasion region from Figure 6B, reveal a more elongated morphology of the invading cells, despite the initially rounded morphology of cells within the tumoroid. On the other hand, MDA-MB-231 cells showed mostly single cell invasion escaping the tumoroid rather than a collective invasion behavior, as shown in Figure 6C.

In order to further quantify the position of invading cells, 3D confocal images were taken; example image stacks of the nuclei were then processed and segmented to give the count and positions of all cells in the sample, as shown for MCF-7 bead clusters in Figures 7A–C. In these images, individual cells have all been assigned a different color. From these three data sets, multiple parameters can be derived to characterize the invasion: The estimated number of cells still inside the bead (*n*_*b*_), the estimated number of cells that have invaded (*n*_*i*_), and the maximum invasion distance (*d_max_*). The position of the original bead is estimated based on the average position of all cells in the sample, and its size is based on the measurement in Figure 3D. Based on the estimated bead dimensions, cells are either considered as inside the bead, or having invaded. In these quantitative analyses (and those reported in Figure 8), migration in all directions was taken into account, i.e., the underlying analysis was based on the full 3D image stack, and we determined cell positions in 3D. Clear differences are found between the three samples: The total number of cells in each sample increases from Figures 7D–F, along with the number of invaded cells, increasing from 9, to 14, to 44 respectively. The percentage of the cells that has invaded also increases with increasing cell numbers, from 14, to 15, to 44%, as well as the maximum invasion distance, from 16, to 50, to 67 µm. We hypothesize that these differences in invasion are related to the initial cell number inside the beads. For the beads in Figures 7E, F, we know that their initial number of cells was different, and that the cells invaded sooner in the sample with the most cells.

### 3.5 Cancer cell invasion in heterogeneous ECM compared to single-ECM models

In order to further assess the cell migration behavior in our heterogeneous cancer model (Figure 8A) in comparison to the conventional single-ECM models, we also seeded microtumors in control experiments using Matrigel (Figure 8B; Supplementary Figure S3) and collagen I only (Figure 8C; Supplementary Figure S4), and characterized the maximum invasion distance (*d_max_*) for all cases (see Figure 8D). To eliminate the initial cell number effect in these comparative studies, the *d_max_* was measured after 3 days in heterogeneous models, and after 2 days in single-ECM models. The extra 1 day in our heterogenous model was enough for cells to fill in the 100 µm bead. In all three conditions, MDA-MB-231 cells invaded further from the initial tumor than the MCF7 cells. The average value of *d_max_* in our heterogeneous model was larger than that of the homogeneous Matrigel but lower than the homogeneous collagen I models for both cell types. For MCF-7 microtumors cultured in Matrigel only, the invasion was virtually absent in comparison with the heterogeneous and collagen I models. This is consistent with previously reported invasion studies based on MCF-7 spheroids in Matrigel. The obvious differences between the *d_max_* in the three ECM models were statistically significant, for both cell types, underlining the importance of integrating correct ECM properties for cellular invasion studies. A further *post hoc* analyses based on the Tukey-Kramer test revealed the maximum invasion distance of MDA-MB-231 in collagen I (217.60 ± 28.96 µm) was significantly larger than in our heterogeneous model (148.40 ± 32.81 µm) and in the Matrigel (150.80 ± 19.32 µm). The same analysis for the MCF-7 cells showed that the measured maximum penetration distance in the three ECM models, i.e. our heterogeneous model (45.20 ± 18.57 µm), Matrigel (12.60 ± 8.70 µm), and collagen I (83 ± 11.70 µm), were significantly different. The multicellular, collective behavior observed for MCF-7 cells in collagen I and in our heterogeneous model, is consistent with behavior reported previously (Barcus et al., 2015; Truong et al., 2016; Zhang et al., 2016; Hayn et al., 2020; Ilina et al., 2020). We observed single cell invasion behavior in all three ECM models for MDA-MB-231 cells, but both the maximum invasion distance (Figure 8D) and the number of invading cells (Figure 8E) depended on ECM conditions. The average number of single invading cells in our heterogeneous model was smaller than for the collagen I model, but two times larger than for the Matrigel model. The number of single invading cells in Matrigel was significantly smaller than in our heterogeneous model (*p* < 0.05) as well as in collagen I (*p* < 0.001), based on two-tailed Student’s *t*-test. We derived the same statistical conclusion using the further *post hoc* analyses (Tukey-Kramer test).

## 4 Discussion

The method presented here enables the generation of a representative, heterogeneous matrix for a pre-invasive breast cancer model, which contains both a stromal ECM and intact BM. We showed that cells can be encapsulated in monodisperse Matrigel beads using the chip and temperature regulated holder, and that these beads can be embedded in a 3D collagen matrix via a sandwiching approach. In experiments with breast cancer cells, we clearly observed microtumor formation and collective penetration and migration for MCF-7 cells, and single cell invasion for MDA-MB-231 cells into the surrounding stromal matrix. In addition, we could quantitatively analyze the microtumors using automated image processing to characterize their invasive state. Finally, we showed that for both MCF-7 and MDA-MB-231 cells, the behavior observed in our hetereogeneous model was significantly different from that in conventional single-ECM models. These promising results indicate that this method is useable for studying breast cancer cell–matrix interactions, with the added advantage of high-throughput generation of many similar microtumors using few manual steps. When combined with methods to vary matrix properties such as stiffness, morphology, and alignment, imposing biochemical gradients such as of oxygen (Sleeboom et al., 2018a), inclusion of other stromal cells, or other types of cancer cells, it has the potential to unlock new discoveries in cancer invasion: the onset of metastasis.

Using our heterogeneously patterned invasion model, we have observed different kinds of behavior ranging from single-cell invasion to collective migration. This indicates that the model can potentially be used to investigate invasion modes as a function of both cancer cell type and the ECM composition. Our comparative study between our droplet-based heterogeneous model, and control experiments using single Matrigel and collagen I models (Figures 8A–C), represented significant differences in maximum invasion distance for both MDA-MB-231 and MCF-7 cells (Figure 8D). When tumoroids were cultured in collagen I, the cells invaded a longer distance, possibly because of micro-tracks present between collagen fibers [Gritsenko et al. (2012), Beunk et al. (2022)]. The invasion distance was smallest for microtumors seeded in Matrigel, likely because Matrigel recapitulates the basement membrane, which needs to be degraded or mechanically broken down to enable invasion onset (Horejs, 2016; Chang et al., 2017; Chang and Chaudhuri, 2019). In our more *in vivo*-like heterogeneous ECM model, we observed larger invasion distances than in Matrigel only, and smaller invasion distances than in collagen I only. This effect was more distinct for the epithelial like MCF-7 cancer cells, exhibiting collective behavior in collagen I and in the heterogeneous model, while the maximum migration distance of MCF-7 cells in Matrigel was negligible in comparison with that in the heterogeneous ECM and the collagen I models. Even though MCF-7 cells are not able to proteolytically remodel ECM since they lack expression of (MT1-MMP)/MMP14, they can penetrate in collagen I by mechanically ‘remodeling’ ECM and pushing themselves through the collagen by their collective mechanical action; for this collective action, their positive E-cadherin status, leading to strong cell-cell junctions, is essential (Ilina et al., 2020). Importantly, the cells move through low-density, compliant inhomogeneities in the matrix; the full-Matrigel environment may be too dense and homogeneous for this to happen, while the collagen facilitates this process. For our heterogeneous model, the cells need to break through the thin Matrigel-collagen I interface at some location(s) and only then penetrate slowly in the surrounding collagen by forming a train of cells, which explains the shorter invasion distance for the heterogeneous model. The leading edge of the penetrating MCF-7 cells does not show signs of migratory phenotype such as filopodia formation, which suggests that this process is driven by physical pushing and penetrating the surrounding matrix, rather than by active collective invasion in which the cells in the migrating front have (at least partially) undergone EMT, and migrate using integrins and proteases (Krakhmal et al., 2015). In future investigations, a comprehensive examination of basement membrane components, such as laminin and collagen IV, and their composition in relation to the dynamics of cancer invasion, may offer insights into the unresolved question of whether the basement membrane undergoes biochemical degradation or mechanical breakdown during the initial stages of tumor invasion. Also, other mechanisms potentially causing the observed differences between the heterogeneous model and the single-ECM models could be examined, such as the possibility that the Matrigel bead mimicking the BM envelope forms a barrier to oxygen or nutrient supply to the cells or to cellular waste products leaving the bead. In addition, our heterogeneous model could be applied to study pre-invasive states of cancer cells, by tracking tumoroids before the onset of invasion (Figures 5I–K for MCF-7; before the 9-h time point shown in Figures 5A for MDA-MB-231), possibly using a more sophisticated imaging system and live staining technologies.

Moreover, in future research normal breast epithelial cells, such as MCF-10A cells, can be encapsulated in Matrigel beads to mimic the healthy mammary gland condition. This use of normal cells can serve two purposes. First, it forms a control experiment for comparison with the cancer cell invasion experiments. The expectation is that the normal cells do not invade, however such a control experiment should be interpreted carefully, since even MCF-10A cells are known to branch/invade collagen I rich matrices; for example, in mixed matrix (Matrigel + collagen I) MCF-10A cells were shown to have a branching phenotype (Qu et al., 2015) and Matrigel/collagen ratio affects the invasiveness of MCF-10A cells (Carey et al., 2017). As an example, Supplementary Figure S5 shows images of MCF-10A spheroids cultured in Matrigel, which did grow in size or grow lobular extensions, but they did not exhibit invasion like we report for the cancer cells. Second, the inclusion of normal breast epithelial cells can bring about the generation of the BM underlying polarized healthy epithelium, as shown in Figure 1A. In this study, we concentrated on the starting point of DCIS via the encapsulation of MCF-7 or MDA-MB-231 cells, mimicking the *in vivo* situation depicted in Figure 1B, where cancer cells have completely filled the lumen but with the BM still intact. Co-encapsulation of normal epithelial cells (for example MCF-10A) and cancer cells in Matrigel beads, would potentially provide a useful model for studying the development of DCIS itself, starting from the healthy gland. This might enable one to study the BM integrity when cancer cells interact with the healthy epithelium and track the cancer cell invasion through the BM generated by epithelial cells. Even though Bischel et al. utilized a Cancer-on-Chip model to develop a DCIS model via lining MCF-10A cells in a duct and then seeding MCF-10A-DCIS cells in the lumen (Bischel et al., 2015b), there are not enough high throughput *in vitro* models for investigating the transition step from pre-invasive DCIS to an invasive cancer state.

Due to the modular nature of our model, the properties of the BM and ECM can be varied independently, and stromal cells such as fibroblasts can easily be added to the stromal ECM. Also, MSCs and ECs could be co-encapsulated with the cancer cells, to achieve a model similar to the STEM model of Li et al. (2022), but with a controlled heterogeneous ECM. Apart from applications in studying the onset of invasion in clusters of breast cancer cells, this method could be applied in a number of different ways. It could for example be combined with laser-ablation techniques to locally modify the ECM and study the effect of local mechanics or even microtracks on invasion Balcioglu et al. (2016); Beunk et al. (2022). Or it could be combined with approaches to induce interstitial flow or techniques to include blood vessels in the matrix Pollet and den Toonder (2020), to mimic cellular waste removal, oxygen and nutrient supply, or even to study intravasation. As mentioned in the introduction, many other tissues contain epithelium and BM, making this pre-invasive model relevant in other contexts as well. In addition, the model could be developed further to investigate particular cancer cell sub-populations. Currently, the cell encapsulation process is geared towards encapsulating multiple cells, but with aggregation preventing adaptations to the protocol, single-cell encapsulation might be achieved. This would open up other applications, such as screening for the tumorigenic and invasive potential of single cancer stem-cells, or even isolated circulating tumor cells.

The read-out and analysis of our model can be further enhanced. Currently, the classification of invaded cells in confocal images of our model is based on an estimated bead position. Ideally, one would measure the exact position of the bead in order to more accurately classify the cells. This could be achieved by staining of the Matrigel beads, or the collagen I matrix, as in Figure 4D. The invasion model would also benefit greatly from incorporation of more targeted detection of, for example, proteins related to the epithelial to mesenchymal transition (EMT), which is thought to be involved in invasion.

Several steps in the model fabrication approach can be improved. We showed that cell sedimentation and aggregation can skew the encapsulation efficiency, which can be addressed in a number of ways. The most straightforward way to reduce sedimentation is to reduce the encapsulation time by increasing the flow-rates. Sedimentation can also be countered by adjusting the fluid density so that gravity and buoyancy acting on the cells are in balance; however, in our case the fluid is Matrigel and adjusting its density would interfere with the properties of our model. Other ways of preventing sedimentation include magnetic stirring in the injecting syringe (Köster et al., 2008), and tip-loading (Sinha et al., 2019), which uses an oil driven pipette tip to inject cells directly in the chip. For these two methods, some modifications to the platform are necessary to ensure that the liquid Matrigel is always cooled. Aggregation is not necessarily an issue for microtumor formation, but it could be minimized to also reduce sedimentation, as larger clusters tend to sediment faster. Cell aggregates could for example be broken up using microfluidic approaches, such as by integrating channels with a micro-pillar array (Park et al., 2014), splitting and merging side channels (Kim, 2015), or periodic constrictions (Qiu et al., 2018).

Apart from sedimentation and aggregation, the limitations of Poisson statistics always result in a spread in the number of cells per bead and the presence of empty beads. Although the empty beads do not interfere with the invasion process, a more controlled cell number per bead would simplify the analysis. This can either be addressed by pre-encapsulation cell ordering, or post-encapsulation bead sorting. Several pre-encapsulation ordering methods exist, based on inertial flow effects (Edd et al., 2008; Kemna et al., 2012; Schoeman et al., 2014) or geometric constraints (Abate et al., 2009). However, these are all dependent on cell size, and therefore not well suited for cell ordering of heterogeneous cell suspensions, especially if there are larger aggregates. Post-encapsulation sorting can be achieved using a combination of optical detection and on-chip actuation, as demonstrated for droplets containing labelled cells (Mazutis et al., 2013; Wu et al., 2013). The downsides of this method are that an advanced optical setup is required, and the cells have to be modified or labeled fluorescently. An alternative method relies on density differences between beads with different numbers of cells, which can be sorted in a standing acoustic wave (Nam et al., 2012).

Some additional improvements can be made to the recovery and embedding method. During the recovery step, many beads are attached onto the paper filter, and are lost from the sample. This does not affect the beads that are deposited on the collagen, and subsequent microtumor development, but it does lead to bead and cell losses in the process. This could possibly be addressed by employing other bead recovery methods, such as double emulsification and spontaneous release (Choi et al., 2016). The key challenge here is to release the soft beads without damaging them. Additionally, despite having control over the z-position by sandwiching, the x/y-position is not controlled. The beads sediment on the first collagen layer in random locations, but for microtumor development, a better spatial bead distribution would be useful. In addition, standardized positioning would simplify imaging and analysis. In future work, this could possibly be achieved by incorporating micro-well structures in the collagen I gel, which only fit a single bead. Collagen I patterning with a flexible stamp has already been successfully used for patterning gut matrices (Wang et al., 2017), and even tumor-stroma cell interaction models (Yue et al., 2018).

## Data Availability

The original contributions presented in the study are included in the article/Supplementary Material, further inquiries can be directed to the corresponding author.
