# Supplementary material for: An in vitro model of cancer invasion with heterogeneous ECM created with droplet microfluidics
Source: Front Bioeng Biotechnol. 2023 Nov 23;11:1267021. doi: 10.3389/fbioe.2023.1267021 (PMC10702358; doi:10.3389/fbioe.2023.1267021)
Supplement: Supplementary file 1 [file DataSheet1.pdf]

Supplementary Material

1 SUPPLEMENTARY VIDEOS

1.1 Videos

Supplementary video 1. A Matrigel encapsulated MCF-7 cell cluster embedded in colagen I, followed during 4 days. During the first 2 days, the cells proliferate to fill the Matrigel bead. Subsequently, cells break through the Matrigel-collagen I interface and form a protrusion penetrating into the surrounding collagen I.

2 SUPPLEMENTARY FIGURES

2.1 Figures

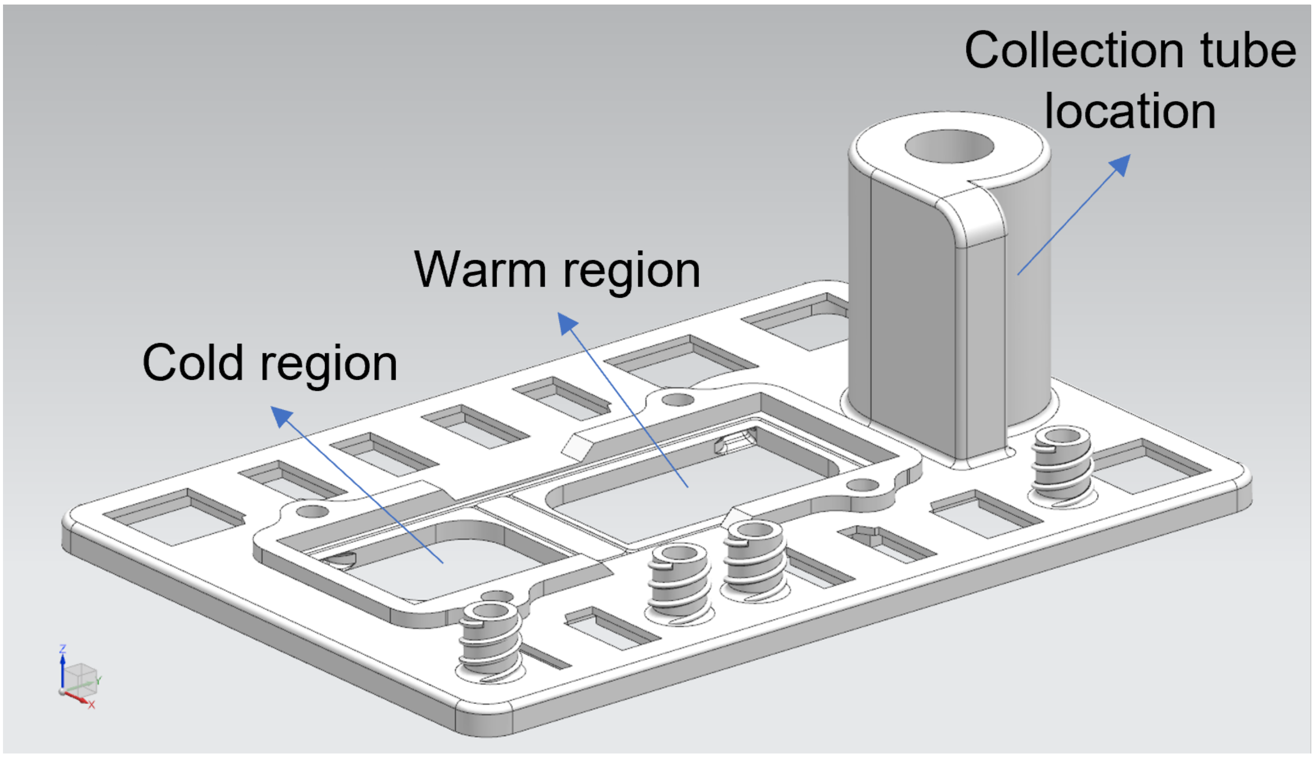

Figure S1. Schematic representation of the 3D printed part of the temperature control platform.

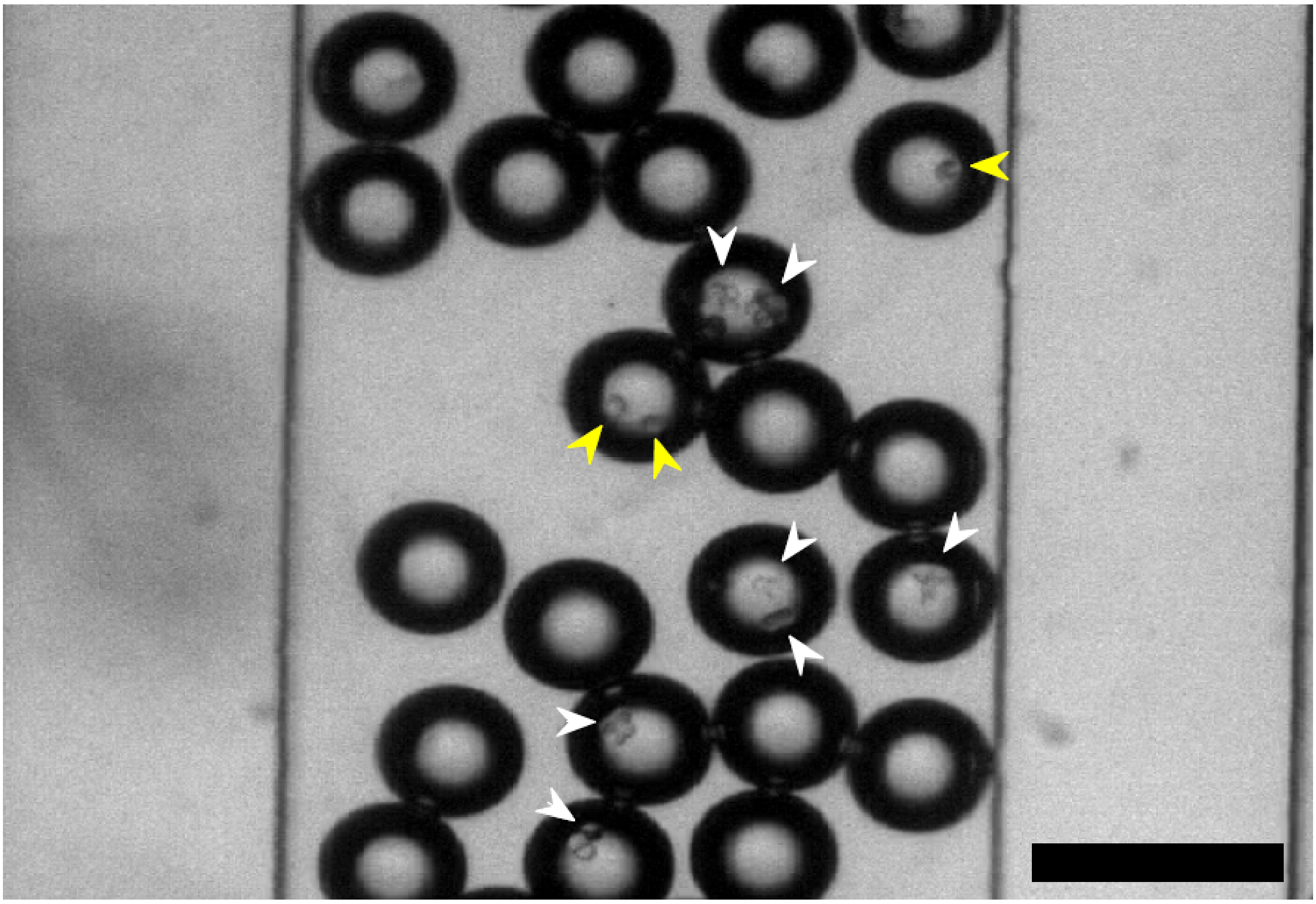

Figure S2. Image of Matrigel beads flowing in the meandering channel of the droplet-maker device. The yellow arrows indicate single cells, and the white arrows indicate clusters. Scale bar: 200 µm.

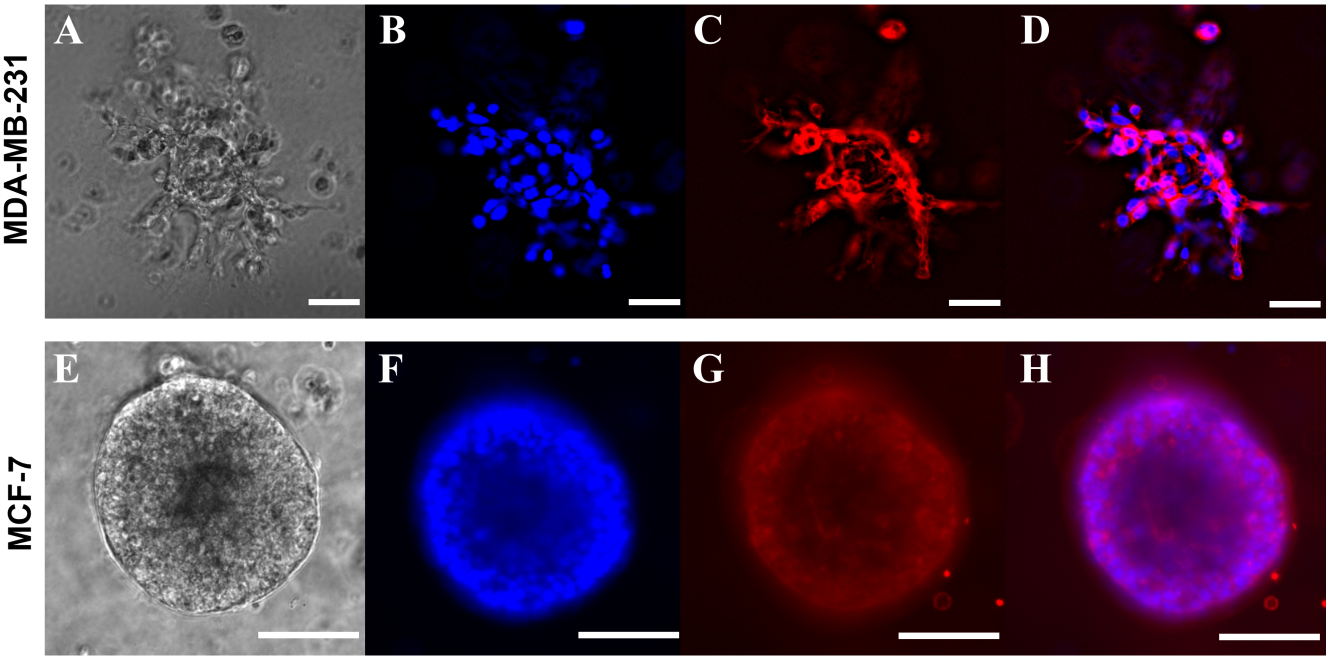

Figure S3. Images of the MDA-MB-231 **(A-D)** and MCF-7 **(E-H)** tumoroids that were cultured in Matrigel for two days. Cell nuclei are stained in blue and F-actin in red. **(A, E)** phase contrast, **(B, F)** cell nuclei, **(C, G)** F-actin, **(D)** merged images in **B** and **C**, and **(H)** merged images in **F** and **G**. Scale bars: 100 µm.

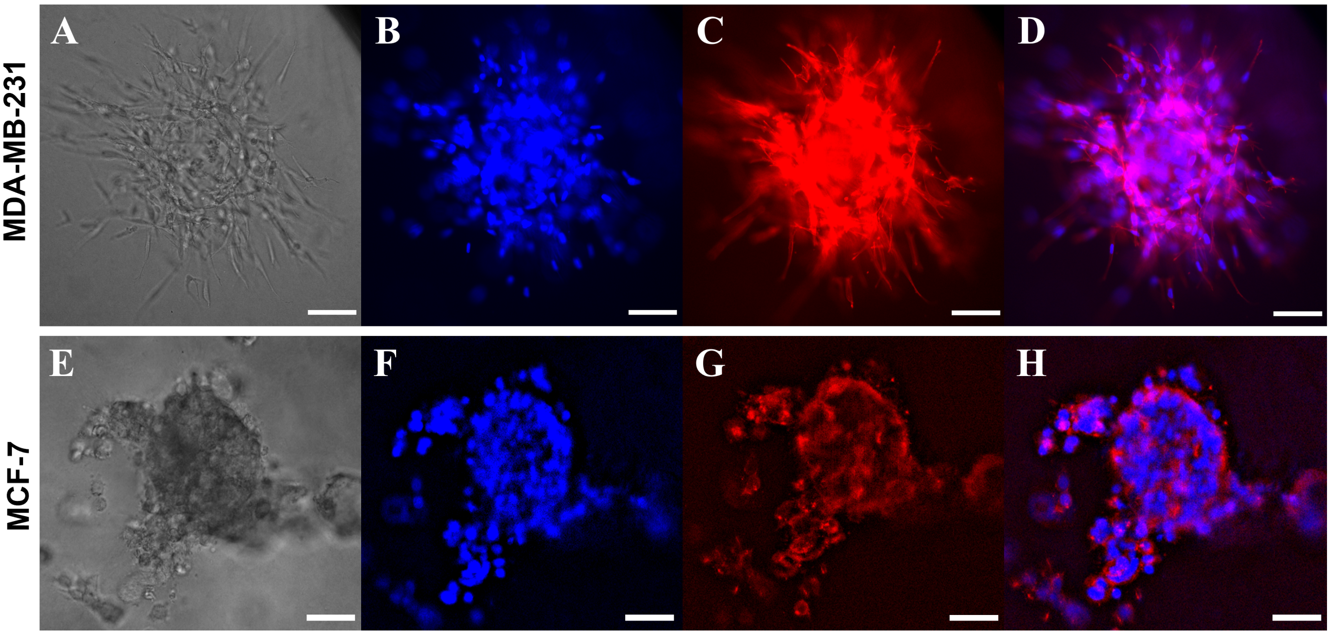

Figure S4. Images of the MDA-MB-231 **(A-D)** and MCF-7 **(E-H)** tumoroids that were cultured in collagen type I for two days. Cell nuclei are stained in blue and F-actin in red. **(A, E)** phase contrast, **(B, F)** cell nuclei, **(C, G)** F-actin, **(D)** merged images in **B** and **C**, and **(H)** merged images in **F** and **G**. Scale bars: 100 µm.

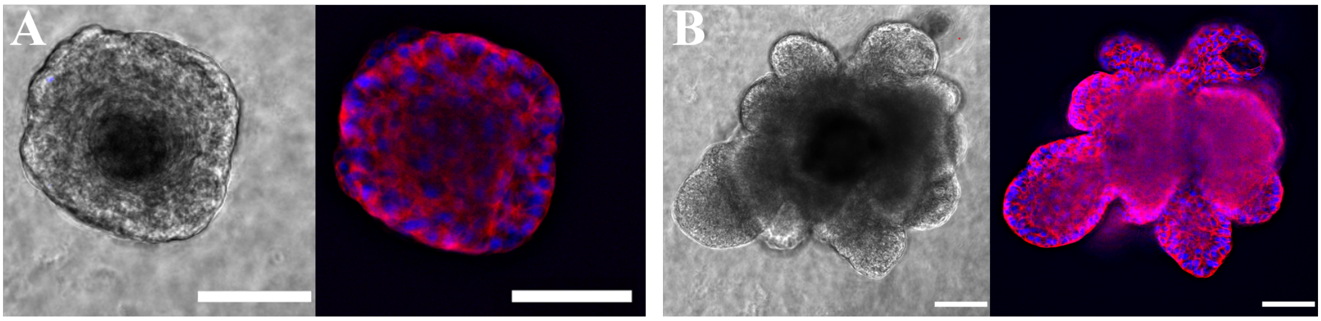

Figure S5. Images of the MCF-10A spheroids that were cultured in Matrigel for six days. The spheroids of normal human breast epithelial cells either **(A)** grew in spherical shape, or **(B)** grew lobular extensions, but did not exhibit migratory behavior like the cancer cells as reported in the main manuscript. **(A, B)** Left: phase contrast image, and right: cell nuclei stained in blue and F-actin in red. Scale bars: 100 µm.
